# Embodiment in 18th Century Depictions of Human-Machine Co-Creativity

**DOI:** 10.3389/frobt.2021.662036

**Published:** 2021-06-28

**Authors:** Anna Kantosalo, Michael Falk, Anna Jordanous

**Affiliations:** ^1^ Department of Computer Science, School of Science, Aalto University, Espoo, Finland; ^2^ School of English, University of Kent, Canterbury, United Kingdom; ^3^ School of Computing, Cornwallis South, University of Kent, Canterbury, United Kingdom

**Keywords:** human-machine co-creativity, embodiment, creativity, design fiction, literature, digital humanities, computational creativity

## Abstract

Artificial intelligence has a rich history in literature; fiction has shaped how we view artificial agents and their capacities in the real world. This paper looks at embodied examples of human-machine co-creation from the literature of the Long 18th Century (1,650–1,850), examining how older depictions of creative machines could inform and inspire modern day research. The works are analyzed from the perspective of design fiction with special focus on the embodiment of the systems and the creativity exhibited by them. We find that the chosen examples highlight the importance of recognizing the environment as a major factor in human-machine co-creative processes and that some of the works seem to precede current examples of artificial systems reaching into our everyday lives. The examples present embodied interaction in a positive, creativity-oriented way, but also highlight ethical risks of human-machine co-creativity. Modern day perceptions of artificial systems and creativity can be limited to some extent by the technologies available; fictitious examples from centuries past allow us to examine such limitations using a Design Fiction approach. We conclude by deriving four guidelines for future research from our fictional examples: 1) explore unlikely embodiments; 2) think of situations, not systems; 3) be aware of the disjunction between action and appearance; and 4) consider the system as a situated moral agent.

## 1 Introduction

Tools for assisting creativity are becoming more commonplace. New systems utilizing artificial intelligence (AI) methods to empower the tools themselves to be creative are stepping in different fields, including robots for playing music (Hoffman and Weinberg, 2010; Weinberg et al., 2020) and singing (Miranda, 2008), sketching (Lin et al., 2020) and even fostering creativity in children (Ali et al., 2019). These co-creative robots represent technological progress in machine engineering, artificial intelligence as well as human-machine interaction.

The idea of machines assisting creativity precedes the current practical advancements by hundreds of years. Simple tools such as musical dice were invented in the 18th Century to enable musically ignorant persons to take part in writing music and received huge popularity in the contemporary intellectual climate fuelled by rationalism (Hedges, 1978). Provided with a pre-composed set of musical fragments, typically musical phrases that fit certain melodic or harmonic constraints, people could construct coherent musical compositions by using dice rolls to select fragments from the set. These musical dice games have acted as inspirations for more modern AI based approaches to interactively compose music with computers (see e.g., Lin et al., 2015).

In this paper we explore examples of co-creative systems from Eighteenth-Century literature in different areas of artistic creativity. We have focused on robotic and other physical systems to emphasize the embodied aspects of human-machine interaction. Like the example of musical dice games we expect these systems to fuel the imagination of modern-day researchers. In the same way that present-day design fictions combine science fiction and design research (Sterling, 2005; Bleecker, 2009; Dunne and Raby, 2013; Blythe, 2014), these older fictions depict potential solutions for solving complex creativity related problems and offer us a new perspective for questioning our design solutions and research approaches. Bruce Sterling, inventor of the term “design fiction”, argues that design fictions aim to “suspend disbelief about change” (quoted in (Blythe, 2014, p. 3)). By taking old speculations about human-machine co-creation seriously, we may discover that new kinds of human-machine co-creation are possible.

## 2 Materials and Methods

This work aims to develop our understanding of embodied creativity, by increasing our knowledge of how human-machine co-creativity has been understood in the past. We analyze five examples, selected by our literary expert author as representative of human-machine co-creativity in the Long 18th Century (c. 1,650–1850): two Romantic poems about aeolian harps (harps played by the wind): Samuel Taylor Coleridge’s “The Eolian Harp” (1795, rev. version 1817, in Coleridge and Keach (1997)) and Eduard Mörike’s “An eine Äolsharfe” (1837, in Mörike (1838)); E. T. A. Hoffman’s “Automata” (1814), a tale containing a multitude of automatic musical systems and a humanoid question/answer machine; the creativity thinking aids featured in Laurence Sterne’s novel *Tristram Shandy* ([1759–67] 2009); the self-conscious Hackney Coach from Dorothy Kilner’s *Adventures of a Hackney Coach* (1781); and the artificial man Homunculus in Goethe’s play *Faust: Der Tragödie Zweite Teil* (1832). Like contemporary design fictions, these literary design fictions take a variety of forms (poetry, fiction, drama).

We have chosen to focus on the Long 18th Century (c. 1650s–1850s) because it marked a watershed in the history of AI (Riskin, 2016). At the beginning of this period, René Descartes set the question of AI on a new footing with his mind-body dualism; by the end of this period, Mary Shelley’s *Frankenstein* (1818) had spawned a powerful myth that still dominates the way AI is imagined today. In between Descartes and Shelley there was a period of great imaginative freedom, when authors experimented with many different kinds of fictional AIs.

Descartes had shattered the Thomistic consensus that mind and matter were interfused, and that only God could create new forms of life. He argued that the body was a mere machine, and that functions of life such as ingestion and sense-perception could be explained by the mechanical workings of matter; the soul was utterly separate from the body, and was responsible for abstract thought alone (Descartes, 1988, 64–65). Later in the period, radical materialists such as Julian Offray de La Mettrie (1996) would dispense with the soul, arguing that thought was also just a function of the body’s machine. These arguments made it possible to believe that scientists might create a living or intelligent machine using the laws of physics alone, and fired the imaginations of writers and inventors alike. In our chosen examples, all sorts of objects are imagined as potentially intelligent: from bowling-greens and Pentagraphs to harps and hackney-coaches. These visions of AI can seem strange and eccentric to the twenty-first-century reader. This is precisely why they are worth considering.

By the end of the Long Eighteenth-Century, speculations about AI had become commonplace, and the marvellous automata that had dazzled the European public had begun to lose their allure for an intellectual or scientific audience (Hankins and Silverman, 1999, 213–216). With her blockbuster novel *Frankenstein*, Mary Shelley simultaneously revived public interest in AI and sent the discussion in a new direction. The myth of the rebellious superintelligence was born. In the 19th Century, novelists such as Samuel Butler (1872) and George Eliot (1879) extended Shelley’s ideas about how AI might evolve beyond its human creators. In our own time, AI theorists such as Kurzweil (2006), Bostrom (2014), Tegmark (2018) and (Russell et al., 2019) have attempted to bring the *Frankenstein*-myth into the scientific mainstream (Falk, 2021). Meanwhile rebellious super-intelligent AIs remain a staple of contemporary science fiction. By looking back to the Long 18th Century, before the *Frankenstein*-myth set in, we hope break open the scientific imagination, and open up new ways of thinking about the roles AI might play in human life.

In our search for new ways of thinking about the roles of AI, we choose to focus on human-machine co-creativity. In this paper human-machine co-creativity is considered as a collaborative activity between a human and a machine driven toward an artistic goal. In human–computer co-creativity, co-creation is often understood as the creation of artifacts via the interaction of different initiatives (Yannakakis et al., 2014), sharing of creative responsibility (Kantosalo et al., 2014) or blending the machine into the human creative process (Davis, 2013). The term encompasses various different ways of organizing the co-creative process and the human and the machine can play different kinds of roles (for example, see the classifications of such roles by Kantosalo and Jordanous (2020); Lubart (2005)) or contribute to the creative process in different ways (Kantosalo and Takala, 2020). The style of human–computer co-creative interfaces is often similar to mixed-initiative interaction (Allen et al., 1999; Horvitz, 1999) for this reason, human–computer co-creativity is sometimes referred to as mixed-initiative co-creativity (Yannakakis et al., 2014) and the related interfaces as mixed-initiative creative interfaces (Deterding et al., 2017). In general human–computer co-creative systems can express various degrees of co-creative intent on a spectrum from full human intent to full computational intent (Deterding et al., 2017).

The selected fictitious examples are analyzed as design fictions for variations in levels of embodiment and creativity. Embodiment was selected as a perspective, since it builds a bridge between contemporary artificial intelligence research and eighteenth-century philosophy. Eighteenth-century scientists such as Jacques de Vaucanson and Wolfgang von Kempelen argued that the achievement of AI would require simulation of bodily systems (Riskin, 2003; Riskin, 2016). These arguments appeared to be justified at the time by extraordinary advances in the design of mechanical calculators, in the construction of “automata” (clockwork robots that replicated bodily movements), and in fields such as optics and acoustics. Meanwhile philosophers such as Étienne Bonnot de Condillac (1984) argued that cognition of external objects would be possible only for an embodied being with a sense of touch. These eighteenth-century arguments foreshadow debates in phenomenology, cognitive science and neuromorphic computing today. It was a period of considerable speculation about the possibility of artificial intelligence, and the literature of the period may contain useful lessons for scientists today.

### 2.1 Selected Works for Analysis

As shown in Table 1, the examples chosen for this work represent a variety of co-creative systems ranging from the very tool-like systems presented in *Tristram Shandy* (Sterne, 2009), through to more genuinely co-creative examples such as the mechanical musicians in E. T. A. Hoffman’s “Automata” (1814) or the almost autonomous Hackney Coach in (Kilner, 1781). The examples also represent different kinds of embodiment, from the utterly non-human aeolian harps in Coleridge and Mörike’s poems (Coleridge and Keach, 1997; Mörike, 1838), to Hoffman’s humanoid Talking Turk (Hoffman, 1957), and the essentially disembodied Homunculus in Goethe’s *Faust* (Goethe, 1832).

**TABLE 1:** Embodied interactions in each example text.

Work	Artificial agent	Agent’s embodiment	Human’s embodiment
Coleridge’s “Eolian Harp” (1795)	An aeolian harp	String instrument played by the wind	The human poet (Coleridge) interacts using sense of hearing
Mörike’s “An eine Äolsharfe” (1837)	An aeolian harp	String instrument played by the wind	The human poet (Mörike) interacts using sense of hearing
Goethe’s *Faust II* (1832)	Homunculus	Tiny artificial human enclosed in a glass phial; glows and can read minds	Human characters converse with Homunculus or have their minds read
Hoffman’s “Automata” (1814)	The Talking Turk	Clockwork fortune-teller with power of speech and mysterious inner mechanism	Human characters ask the Turk questions, hear its answers and peer into its mechanism
	Artificial performers	When activated by a human, the performers create music	Humans activate the artificial performers, play alongside them, and listen as the audience
Kilner’s *Hackney Coach* (1781)	A hackney coach	Coach can see and hear its immediate surroundings, but does not control its own movements	Humans drive or ride on the coach, unwittingly providing the material for its narrative
Sterne’s *Tristram Shandy* (1759–67)	Mechanical writing-aides	Audio or optical devices that change the appearance of an observed object	Human narrator imagines using different aides to gain different insights into his human characters
	The bowling green	A bowling green of soft earth that is shaped into a scale model of key battlefields in the nine years’ war	Human characters manually update the bowling green model as news arrives from the front

The first two examples are the aeolian harp poems of Samuel Taylor Coleridge and Eduard Mörike. Aeolian harps were popular stringed instruments of the 18th and 19th centuries. They could take various forms, but all aeolian harps were harps designed to be played by the wind rather than human fingers. They were of particular interest to early researchers in acoustics, who were perplexed by the harps’ peculiar creative properties: when a harp’s string is plucked by a human, it can only produce one note, but when played by the wind, it can produce a great variety of different notes (Hankins and Silverman, 1999, ch. 5). Poets like Coleridge and Mörike were also interested in the harps’ creative properties, but interpreted the harp in a more mystical way. Coleridge, for instance, seems to have believed that the harp, the wind, and its human listener all participated in a shared consciousness:

And what if all of animated nature

Be but organic Harps diversely fram’d,

That tremble into thought, as o’er them sweeps,

Plastic and vast, one intellectual Breeze,

At once the Soul of each, and God of all? (Coleridge and Keach, 1997)

In Mörike’s poem, there is a similar ambiguity. As the poet listens to the harp, it is hard to distinguish whether the emotions of the music are the harp’s emotions, the poet’s emotions, or emotions that are latent in the situation as a whole. We see these as poetic examples of extended consciousness.

In Part Two of Johann Wolfgang von Goethe’s *Faust*, we encounter a more typical fictional AI: the creature Homunculus. Homunculus is an artificial man created by Wagner, a scientist and alchemist. What makes Homunculus unusual, especially when compared to more famous fictional AIs such as Frankenstein’s monster, is his embodiment. His human body is minuscule, and contained within a fragile glass phial. He doesn’t seem to make any use of his human body parts. Instead of walking, he floats in mid-air. He also has the ability to glow, read human thoughts, and later, absorb himself into other beings. In some regards, Homunculus resembles a disembodied software program more than an embodied human, despite his human appearance, and looks forward to the disembodied AIs of cyberpunk classics such as Masamune (2009); Gibson (2016).

The artificial agents in E.T.A. Hoffman’s story, “Automata”, are more down-to-earth, because they are based on actual automata that were built and exhibited in Eighteenth-Century Europe. At the beginning of the story, the main characters encounter the Talking Turk, a clockwork question-answering system of stereotypically Turkish appearance. The Turk answers users’ questions, and is cleverly designed to defy users’ attempts to work out how it operates. The other automata in the story are a group of clockwork musicians, based on some famous examples by the French artificer Jacques de Vaucanson (Riskin, 2016, ch. 6). The main characters meet the creator of these musicians. The creator turns them on, and plays a piece with them on his piano. One useful feature of this example is that the main characters disagree about whether any of the automata are truly intelligent or creative.

Dorothy Kilner’s *Adventures of a Hackney Coach* (1781) is an “it novel” or “novel of circulation”. This was a popular genre of fiction in the late 18th Century, in which an inanimate character such as a coin, atom or statue would narrate their adventures in the world (Bellamy, 2007). In Kilner’s novel, the narrator is a Hackney Coach, who is driven around London and the surrounding area picking up passengers from different social classes. The novel is told from the Hackney Coach’s perspective, as it recounts the conversations of its passengers and describes the different places and events it visits. There are two key creative collaborations: the Coach collaborates with its passengers, who unwittingly provide the material for the story, and the Coach collaborates with the human reader, to whom the narrative is addressed.

Sterne’s novel *Tristram Shandy* (Sterne 2009) is the fictional autobiography of the main character, Tristram Shandy. It is a famously experimental and unusual novel. Although it is ostensibly the story of Tristram’s life, he gets so sidetracked describing the lives of his father, Walter Shandy, and his Uncle Toby, that never manages to narrate more than the first few years of his own life. The book also contains many digressions, where Tristram discusses the art of novel-writing and other mostly irrelevant topics.

*Tristram Shandy* includes two interesting examples of co-creative machines. In Volume 1, Chapter 23 the narrator describes several imaginary writing aids that allow the writer to develop character depictions. Momus’s glass is a device installed on a character’s chest, which enables the writer to perceive the inner workings of the character’s soul as if through a window. Other writing aids include musical instruments that play the characters’ emotions, a Pentagraph, a mechanical device that exactly replicates the movements of the human writer’s pen, and the “Hobby-Horse”, which Tristram uses to reveal a character’s driving obsession. For clarity, we have focused on Momus’ glass, the most extreme and also best-described of Tristram’s imaginary co-creative machines. The second main co-creative system is the bowling green used by Uncle Toby, which Tristram describes in particular in Volume 2, Chapter 1, and Volume 6, Chapters 21–23. The bowling green is a massive model of the Nine Years’ War, and, ironically enough, it is Uncle Toby’s “Hobby Horse”, linking it to the first set of co-creative machines. We look at this model as a physical co-creative environment in which Uncle Toby acts and analyses various events of the war.

### 2.2 Design Fiction

How can two hundred year old fictional texts inform scientific research today? None of the examples we have chosen describe a scientific process by which a creative artificial agent could be made. Indeed, some of the examples are impossible. Dorothy Kilner’s intelligent Hackney Coach, for example, is able to see and hear events in its immediate surroundings even though it apparently lacks any sensory organs, and its personality is notably human and English despite the fact it is a coach. Rather than explaining how this kind of intelligent agent exists, the novel takes the agent for granted and explores its implications.

We therefore propose to interpret these texts as *design fictions* (Sterling (2005); Bleecker (2009); Dunne and Raby (2013); Blythe (2014)). The purpose of design fictions is not to show scientists how to solve a problem, but rather to help scientists determine what problem they should try to solve. Design fictions achieve this purpose by simulating the experience of interacting with new technologies. Instead of describing how a particular technology functions, or describing the process of manufacture, design fictions presuppose that such a technology exists, and portray what interacting with it would be like. A design fiction is typically in the form of a film, novel, poem, play or art installation. When we read or view such a fiction, we imagine ourselves in a new world, where a new technology exists, and can feel what it might be like to live with such a device. We imagine the future ‘from the inside’, and gain an intuitive, embodied, subjective understanding of which technologies we might desire to have or wish to avoid (Burdick, 2019). In this way, design fiction offers two key benefits to scientists: goalposts and warning signs. Goalposts: by painting a vivid picture of humanity’s possible futures with technology, design fiction can fire scientists’ imaginations and widen the search space. Warning signs: by allowing us to explore the “implications” rather than simply the “applications” of new technologies, design fiction can help scientists determine the moral and ethical implications of their research (Dunne and Raby, 2013, p. 49).

These historical texts were not necessarily intended as “design fictions”, but by focusing on the interaction between the human and non-human agents, we can read them as if they were. To read these texts as design fictions, we focus specifically on embodied interaction. How does the embodiment of the fictional artificial agent affect its cognition of the world and its interaction with human beings? And likewise, how does the embodiment of human agents affect their interaction with the artificial agents? In Mörike and Coleridge’s poems, for example, the artificial agent is an aeolian harp. Such harps are large stringed instruments that are played by the wind. Due to this embodiment, they must be placed outside or in a window, where the wind blows, and they produce output in the form of sound. The agent is therefore stationary, under the wind’s influence, and surrounded by outdoor scenes. To interact with the harp, the human agent must go outside, wait for the wind, and listen with their ears. Thus the embodiment of the harp and the human intersect to produce a particular kind of interaction. The poems describe this interaction in vivid language, recreating the experience of listening to the aeolian harp and offering the reader an intuitive understanding of what value such an interaction could have.

### 2.3 Embodiment and Creativity

Viewpoints on embodied creativity and collaboration, and on embodied artificial intelligence more generally, range across a number of notions (Chrisley, 2003; Ziemke, 2015). Competing theories about the role of embodiment in cognition range from theories of minimal embodiment, which look at the embodiment of humans through a reduced body without a brain, to views of embodiment that connect the body with the environment, or allow it to absorb tools to extend its embodiment, and all the way to radical views of embodiment viewing perception as an action oriented concept shaping most cognitive processes (Gallagher, 2011), or behavior-based robotics (Brooks, 1991). Dreyfus (1979) argued that some form of embodiment was necessary for intelligence, echoing the arguments of eighteenth-century materialists such as Condillac and La Mettrie.

Very few works of human–computer co-creativity address aspects of embodiment specifically or take a stance on different theories of embodiment. Embodiment is briefly discussed in some works featuring robotic collaborators, such as an investigation of task-transfer (Fitzgerald et al., 2017), and the works of Saunders, Gemainboeck and their colleagues (e.g., Saunders et al., 2010, 2013; Gemeinboeck and Saunders, 2013), which feature embodied robots that allow for shifting the balance of co-creative initiative toward machine initiative. A few theoretical works, discuss the extended mind theory (Bown, 2015), an enactivist theory for co-creation (Davis et al., 2015) and the wide acceptance of creativity as a situated activity within the field of computational creativity (Guckelsberger et al., 2017), but there appears to be no unified view of how to look at embodiment from the perspective of human–computer co-creativity. Therefore for this paper we wanted to find a disambiguation of embodiment that would more directly address creativity and collaboration with machines.

Dag Svanæs (2013) created a bridge between creativity and embodiment in his work investigating the role of embodiment in interactive technologies. Svanæs applies Merleau-Ponty’s ideas about the lived body and embodied perception into analyzing interaction with technology. As a result he created three concepts “the feel dimension”, “interaction gestalts” and “kinaesthetic thinking” which he used to discuss different kinds of digital products and interfaces. From the last concept he developed the idea of “kinaesthetic creativity”, which discusses how a designer, embedded in a rich context through their lived body can use that experience to create new solutions to problems perceived in that moment.

This paper examines the effects the embodiment of the example systems may have on their creativity. In his paper, Svanæs (2013) shows how to use Merleau-Ponty’s ideas selectively to perform formative analysis of interaction with a few examples ranging from abstract creative art to e-readers. Svanæs’ examples focus on software oriented artifacts with tangible physical interfaces. To be able to compare fictitious robotic examples in a summative, systematic manner, we took the twelve key components Svanæs’ derives from Merleau-Ponty’s (1962) to support his user interaction concepts and turned them into comparison criteria. The twelve criteria are *active perception; perception shaped by the phenomenal field; directed perception; mediating perception through artifacts; whole body perception; the lived body; incorporating artifacts into the body; body schema; bodily space; skills acquisition; the dynamic nature of the body, tools and objects; and concrete and abstract movement*.

Following Svanæs’ (2013) descriptions, the first concept, *active perception*, focuses on human perception as active uses of senses instead of passive reception of stimuli. The second concept, *perception shaped by the phenomenal field*, looks at how the individual background, such as experiences and training, affect human perception. The third concept, *directed perception*, looks at the intentions of the individual affecting what and how they perceive. The fourth concept, *mediating perception through artifacts*, looks how the body can adapt and extend its perceptional capacity through the use of artifacts, such as by a visually impaired person navigating with a stick. The fifth concept, *whole body perception*, looks at how the whole body can be used automatically to extend perceptional capacity, such as turning an object while visually perceiving it to take in various angles. The sixth concept, *the lived body*, considers the body as a general medium of presence in the world. The seventh concept, *incorporating artifacts into the body*, looks at assuming artifacts as part of the lived body, such as a person using a wheelchair. The eight concept, *body schema*, describes the “nonconscious knowledge” an individual has of their lived body and its potential actions in the world. The ninth concept, *bodily space*, considers the degrees of freedom the lived body has in the space. The 10th concept, *skills acquisition*, considers how an individual is able to “internalize external devices through learning”. The 11th concept, *the dynamic nature of the body, tools, and objects*, looks at the changing contextual meaning and purpose of the body, tools and objects. The 12th concept, *concrete and abstract movement*, looks at movements “made naturally as part of a situation” and movements made for the purpose of movement.

We combine this analysis of embodiment with a separate analysis of creativity. This allows us to explicitly consider how embodiment and creativity interact in fictions of this period. In modern creativity research, creativity is characterized by many aspects (Jordanous and Keller, 2016), which vary in importance across domains. Over the years some authors such as Kantosalo and Takala (2020) have attempted to establish frameworks for describing human–computer co-creativity that take into account various theories of human creativity, including for example Glăveanu (2013) and Csikszentmihalyi’s (1988) views of creativity as a socio–cultural act and Glăveanu’s views of material affordances of the creative environment. But to our knowledge, there is no single definition for creativity that is adopted over others in human–computer co-creativity research. Therefore for our analysis we have attempted to find some more general definitions of creativity that would fit the variety of the literary samples examined here.

Creativity research commonly adopts a minimal “bifold” definition of creativity (Runco and Jaeger, 2012), but such a minimal definition makes it difficult to classify the myriad forms of creativity encountered in literary texts. Creativity is an example of an *essentially contested concept* (Gallie, 1955; Jordanous, 2012), in that by its nature, creativity resists full, complete and fixed definition (Corazza, 2016). The nature of creativity has been much discussed from multiple perspectives and various disciplines (Jordanous and Keller, 2016) (e.g., Guilford (1950); Gero (1996); Gabora (2005); Hennessey and Amabile (2010); Weisberg (2015), as a selection of a few different perspectives in a vast and multi-disciplinary area of research).

It is widely acknowledged that practical concerns drive us to adopt working definitions where necessary (Runco and Jaeger, 2012), definitions which others may see as partial or incomplete for their purposes. This paper provides a good example: we argue that the “standard definition” of creativity proposed by Runco and Jaeger (2012) is insufficient for this work, whereas many creativity scholars would find this definition adequate for their purposes. Instead we include in our considerations 14 components of creativity, which were derived by Jordanous and Keller (2016) from computational analysis of a corpus of seminal articles spanning a period of 60 years of research on creativity, from multiple disciplinary perspectives. We do not claim that these components form a conclusive and complete definition of creativity; however for practical purposes these components enable a more divergent, detailed and multi-faceted analysis of the literary and embodied context of this work, considering aspects such as generative ability and originality, as well as social interaction and communication. The components are listed and briefly defined in Table 2.

**TABLE 2:** Components of Creativity, with definitions adapted from Jordanous and Keller (2016).

Component	Definition (adapted from Jordanous and Keller (2016))	
Active involvement and persistence	Being actively involved; reacting to, deliberate	
	Tenacity to persist, even at problematic points
Generation of results	Working toward some end target, or goal, or result
	Producing something that previously did not exist
Dealing with uncertainty	Coping with incomplete, missing or ambiguous information
	Element of risk/chance, lack of routine/pre-existing methods
Domain competence	Domain-specific intelligence, knowledge, expertise
	Recognizing problems and generating new ideas in that domain
General intellect	General intelligence and intellectual ability
	Flexible and adaptable mental capacity
Independence and freedom	Working independently with autonomy over actions/decisions
	Freedom to work, perhaps challenging cultural/domain norms
Intention and emotional involvement	Personal and emotional investment, immersion
	Intention/desire to perform a task, for fulfilment/enjoyment
Originality	A new product, or doing something in a new way
	Results that are unpredictable, unexpected, surprising
Progression and development	Movement, advancement, evolution during a process
	Some developmental progression in a domain/task
Social interaction and communication	Communicating and promoting work to others
	Mutual influence, feedback, collaboration
Spontaneity/Subconscious processing	Thoughts may inform a process subconsciously
	Reacting quickly and spontaneously when appropriate
Thinking and evaluation	Consciously evaluating several options
	Proactively selecting a decided choice from possible options
Value	Making a useful contribution valued by others
	End product is relevant and appropriate
Variety, divergence and experimentation	Generating different ideas to compare and choose from
	Multi-tasking during a process

## 3 Results

We conducted our analysis of the works such that relevant passages of the works would be read by two researchers separately after which the researchers discussed the different elements of embodiment and the different aspects of creativity in the examples.^1^ Based on these discussions we compiled two tables which allowed for comparing and contrasting the different examples through these elements and also to examine whether the embodiment of the systems had any interesting connections to the creative capacities exhibited by the examples (Tables 3, 4). In each case, the question was whether the relevant fictional agent was capable of the given component of embodiment, or the given component of creativity. For example, in none of the fictional examples did the artificial agent acquire a new skill, so all received a null score for “skills acquisition” (Table 3). In some cases, the situation was ambiguous. In Hoffmann’s “Automata”, for example, the Talking Turk seems to display “general intellect” because it is able to listen to and answer any question posed by a member of the public (Table 4). But the story is deliberately ambiguous as to whether the android is actually intelligent or is just a hoax: when the Turk talks and gestures, “die Rückwirkung eines denkenden Wesens unerläßlich schien” [the agency of some intelligent being seemed essential] (Hoffmann, 1957, vol. 6, p. 82). When we felt that an example was insuperably ambiguous in this way, we have placed a “??” in the relevant cell of the table.

**TABLE 3:** Analysis of works by embodiment criteria adapted from Svanæs (2013).

	Mörike, “Äolsharfe”	Coleridge “Eolian Harp”	Kilner, “Hackney Coach”	Sterne, “Tristram Shandy”; bowling green	Sterne, “Tristram Shandy”; writing aides	Goethe, “Faust: Zweiter Teil”	Hoffmann, “Automata”; Talking Turk	Hoffmann, “Automata”; instruments
Active perception	x	x	x	—	—	x	x	—
Perception shaped by the phenomenal field	—	—	x	—	—	x	—	—
Directed perception	x	x	x	—	x	x	??	—
Mediating perception through artifacts	—	—	x	—	—	—	—	—
Whole body perception	x	x	—	—	—	x	—	—
The lived body	—	—	x	—	—	x	—	—
Incorporating artifacts into the body	x	x	??	x	x	x	—	x
Body schema	x	x	x	—	—	—	—	—
Bodily space	x	x	x	x	x	—	x	x
Skills acquisition	—	—	—	—	—	—	—	—
The dynamic nature of the body, tools and objects	—	—	—	x	x	—	—	—
Concrete and abstract movement	—	—	??	—	—	??	—	—

**TABLE 4:** Analysis of works by creativity component.

	Mörike, “Aolsharfe”	Coleridge “Eolian Harp”	Kilner, “Hackney Coach”	Sterne, “Tristram Shandy”; bowling green	Sterne, “Tristram Shandy”; writing aides	Goethe, “Faust: Zweiter Teil”	Hoffmann, “Automata”; Talking Turk	Hoffmann, “Automata”; instruments
Active involvement and persistence	x	x	x	x	—	x	x	—
Dealing with uncertainty	—	—	x	—	—	—	—	—
Domain competence	—	x	x	—	—	—	x	—
General intellect	—	—	x	—	—	—	??	—
Generating results	x	x	x	x	—	x	x	—
Independence and freedom	x	x	??	—	—	x	x	—
Intention and emotional involvement	x	x	x	—	—	x	x	—
Originality	—	—	x	—	x	x	x	x
Progression and development	—	—	—	—	—	x	—	—
Social interaction and communication	x	x	x	x	—	x	x	—
Spontaneity and subconscious processing	x	x	x	—	—	x	x	—
Thinking and evaluation	??	??	x	—	—	x	??	—
Value	x	x	x	x	x	x	x	??
Variety, divergence and experimentation	x	x	??	??	x	x	x	—

## 4 Discussion

Advocates of speculative design argue that design fictions “can play a significant role in broadening our conception of what is possible” (Dunne and Raby, 2013, p.162). Our examples could play this role, by helping scientists rethink core concepts of computational creativity, including embodiment, agency and creativity itself. These texts break down the usual template of creative activity: the human being. They suggest that radically non-human actants such as musical instruments, vehicles or even the ground may exercise certain kinds of creative agency. If scientists engage with these texts, they may rethink their assumptions about the form a creative system might take, invite new analysis of ethical and social implications, and open new lines of inquiry.

### 4.1 Concrete and Abstract Body: Varying Levels of Agency

Most of our examples describe creativity as an automatic or mechanical process, which does not require intellect or self-consciousness. In Table 3, for example, only a few of the examples display “skills acquisition”, a “dynamic relationship between body, tools and objects”, or the distinction between “concrete and abstract movement”. This last component is particularly interesting in the cases of the Hackney Coach and Homunculus. Both of these artificial agents have human-level general intelligence, and are able to interact socially with human beings. The Hackney Coach composes a novel describing its life, while Homunculus converses with other characters. But neither of them seem to distinguish between unconscious “concrete” movement (e.g., the automatic movement of the fingers while typing) and conscious, intentional ‘abstract’ movement (e.g., carefully positioning the fingers to pick up a sharp object). The Hackney Coach moves passively, according to the pulling force of its horse and the direction of its driver. It is therefore capable *only* of “concrete”, unconscious movement, as its wheels turn to accommodate the direction of the horse and driver. Homunculus, by contrast, moves intentionally, but his form of movement (levitation) requires no bodily action. In his case, he is capable of purely “abstract”, intentional movement, but not of “concrete”, unconscious movement. In this way, both these agents break down the distinction between “conscious” and “unconscious”, at least as far as bodily movement is concerned.

This lack of bodily self-consciousness correlates with several components of creativity. Few of the agents “deal with uncertainty”, possess “domain competence”, have “general intellect”, or “progress and develop” (Table 4). Their creativity is generally spontaneous and adventitious, rather than self-conscious and deliberate. In Coleridge and Mörike’s poems, for instance, the aeolian harps create original music, decode emotional information that is encoded on the wind, and communicate it to human listeners who participate by providing the missing ingredient of self-consciousness. Mörike, for instance, describes how the wind:

… säuselt her in die Saiten,

Angezogen von wohllautender Wehmuth,

Wachsend im Zug meiner Sehnsucht,

Und hinsterbend wieder. (Mörike, 1838, p.52)

[… rustles hither in the strings,

Drawn by eloquent melancholy,

Growing in the pull of my desire,

And dying away again.]

The wind appears to feel some emotion, being itself “drawn” to the poet’s melancholy. Meanwhile the poet responds emotionally to the music of the wind in the strings of the harp. The harp creates such music, and communicates such emotion, without apparently having any kind of intellect or consciousness.

This example raises the difficult problem of agency: How can we ascribe creative intentions or actions to the harp or the wind? In his own aeolian harp poem, Coleridge speculates that there is “one life, within us and abroad, — Which meets all motion and becomes its soul” (Coleridge and Keach, 1997, p. 87). If there is indeed such a global “soul”, “life” or consciousness that pervades all things, then it would be perfectly possible for the wind to intend or to act. But such a “world-soul” is scarcely consistent with modern science. According to Riskin (2016, pp. 1–2), in contemporary physics and chemistry it is considered unacceptable to describe any physical system by ascribing agency to its components. Probabilities and causes are acceptable explanations, not decisions. This hesitancy over agency is quite palpable in the field of computational creativity. In a classic definition of the field, Wiggins (2006, p. 2) says that Computational Creativity is:

The study and support, through computational means and methods, of behaviour exhibited by natural and artificial systems, which would be deemed creative if exhibited by humans.

This strange phrase, “would be deemed creative’, indicates an insecurity at the heart of the field. If no agency can be ascribed to “natural and artificial systems”, then how can a computer actually *be* creative? At best it can merely simulate or model creative activity.

This problem of agency, creativity and machinery is addressed explicitly in Hoffmann’s “Automata”, when the characters debate whether the mechanical musicians create genuine music. After hearing the mechanical musicians, the characters Ludwig and Ferdinand come to differing conclusions. Ludwig finds the automatons’ music “zuwider” [repugnant], arguing that the agency of a human musician is required to create true music (Hoffmann, 1957, vol. 6, p. 105).^2^ Ferdinand finds the artificial music beautiful, though agrees that it is inferior to human music. Interestingly, although Ludwig loathes the machines’ music, he nonetheless claims that music has an ultimately nonhuman source:

“Kann denn”, ewiderte Ludwig, “die Musik, die in unserm Innern wohnt, eine andere sein als die, welche in der Natur wie ein tiefes, nur dem höhern Sinn erforschliches Geheimniss verborgen, und die durch das Organ der Instrumente nur wie im Zwange eine mächtigen Zaubers, dessen wir Herr worden, ertönt?” (Hoffmann, 1957, vol. 6, p. 107) [“Can it be,” replied Ludwig, “that the music that lives within us is different to that which lies as a deep mystery in Nature, discoverable only by the highest sense, and which is expressed by instruments only under the compulsion of a mighty spell of which we are the masters?”]

For this reason, although the automaton musicians are not to his taste, Ludwig is more open to the music of Aeolian harps, and claims that a “höhere musikalische Mechanik” [“higher mechanics of music”] is possible (Hoffmann, 1957, vol. 6, p. 105). Thus even this more sceptical, scientific text leaves open the possibility that nature itself may have creative properties that could be harnessed by a mechanical system.

This issue of agency is particularly prominent in *Tristram Shandy*, whose artificial agents lack not only self-consciousness, but also “independence and freedom”, “intention and emotional involvement” and also, in one case “originality” (Table 4). In terms of embodiment, the agents also lack “active perception” and the “shaping force of the phenomenal field” (Table 3). The bowling green is entirely shaped by human hands, and has no sense organs of any kind, while the writing aids, such as Momus’ glass, are essentially optic, acoustic or haptic instruments that provide the writer with novel input about the character they are trying to describe. Momus’ glass provides a vision of the character’s heart, but there is no suggestion that the glass actually *sees* the character’s heart itself.

Nonetheless, in the novel both the writing aids and the bowling green frequently intervene in the plot and change the course of events, displaying a form of material agency which does not require any kind of awareness. In a mundane way, the bowling green’s soil composition affects how well Uncle Toby’s model operates:

Nature threw half a spade full of her kindliest compost upon it, with just so much clay in it, as to retain the forms of angles and indentings—and so little of it too, as not to cling to the spade, and render works of so much glory, nasty in foul weather (Sterne, 2009, p. 342, p. 342).

This is the most obvious way the bowling green’s embodiment affects its collaboration with humans, but throughout the novel, the bowling green acts in other, more surprising ways. It is directly implicated in Tristram’s accidental circumcision, for instance. Having misplaced the chamber-pot, Tristram’s maid Susanna instructs the young boy to “**** *** ** *** ******” (Sterne, 2009, p. 301).^3^ Unfortunately, all the leaden counterweights in Tristram’s house have been resumed by Uncle Toby’s trusty corporal Trim, to be melted down and make cannons for their bowling-green model of the Nine Years’ War. The sash window falls, and the unfortunate Tristram suffers a surprise operation. Admittedly, in this case, the bowling green acts through human agents, whose search for raw materials to upgrade the bowling green is the proximate cause of the accident. But the novel is replete with jokes the blur the boundaries between human bodies and physical objects in their environment. Tristram’s mother relies on hearing the sound of Tristram’s father winding the clock in order to experience sexual arousal (Sterne, 2009, p. 9). Near the end of the novel, when Uncle Toby is wooing the widow Wadman, she is curious about the groin injury he sustained at the siege of Namur. “You shall lay your finger upon the place”, Uncle Toby tells her (Sterne, 2009, p. 514). She is at first surprised by this intimate suggestion, but Uncle Toby then fetches a map, and allows her to lay her finger on the geographical place where he was wounded. The map and the man become thoroughly confused. The confusion is even greater when we consider that Uncle Toby’s bowling green is also the “Hobby Horse” that Tristram uses as a writing aid to describe his Uncle’s character. Tristram jokes that “a man’s HOBBY-HORSE is as tender a part as he has about him” (Sterne, 2009, p. 91). Like a tender body part, Uncle Toby’s bowling green can cause him pain, and Tristram can use it as a creative collaborator to illuminate Toby’s personality.

It is in this broad context, in which human bodies and confused with the material world around them, that the bowling green, and Tristram’s imaginary writing-aids, seem to acquire agency, despite lacking some of the usual embodied and creative components that an agent would be expected to have. In its anarchic, comedic way, *Tristram Shandy* foreshadows contemporary philosophers of science, such as Jane Bennett, who argue that our conventional understanding of agency is too narrow, and that mere matter may have an “agentic power” that we overlook when we define agency in term of human intentionality and consciousness (Bennett, 2010, p. 69). Similar ideas about attributing creative agency to the materials participating in a creative act are also expressed by some computational creativity scholars, such as Bown (2015).

*Tristram Shandy* is not very explicit about where this “agentic power” may come from. The bowling green shifts and morphs, drawing Tristram and Uncle Toby’s bodies into itself; the writing aids somehow create a connection between the writer’s pen and the character’s personality. These processes are joked about rather than explained. Similarly, in *Adventures of a Hackney Coach*, the Hackney Coach simply *is* sentient, with no attempt to explain this surprising fact. Aside from Coleridge, with his idea of the “one life”, the only author in our sample who seriously attempts to put forward a more general theory of creative agency is Johann Wolfgang von Goethe. When Wagner creates Homunculus, he argues that matter has an innate self-organizing tendency which allows it to become creative:

Was man an der Natur Geheimnißvolles pries,

Das wagen wir verst ändig zu probiren,

Und was sie sonst organisiren lie ß,

Das lassen wir krystallisiren (Goethe, 1832, p. 105). [What we thought before was Nature’s secret,

That is what we now dare to experiment with.

And what Nature once allowed to self-organize,

We now allow to crystallise.]

Homunculus is “crystallized” out of a material that already has a natural tendency to organize itself. Even Wagner, the human scientist who is an agent in the usual sense, “lässt” [“lets”] the crystallization occur. As in the case of the Aoelian harps, where the environment itself, the wind, plays a key role in the creative process, in *Faust*, there seems to be no real difference between human agents and inert matter—everything has some kind of “agentic” or “organizing” power, and can collaborate in the creative process.

These texts present a challenging view of creative agency, which could influence the way researchers design creative robotic systems. First, if an object can be a creative agent while lacking intelligence, intention and even perception, then this could impact how we evaluate creative systems (Jordanous, 2017; Kantosalo, 2019). This problem of evaluation, as we have seen, is explicitly raised in Hoffmann’s “Automata’, when Ferdinand and Ludwig dispute the creativity and intelligence of the story’s robotic systems, based on their differing attitudes about the uniqueness of human agency. Secondly, by opening up the field of how a creative agent might be embodied, these texts could inspire new designs. A creative robot could emulate Uncle Toby’s bowling green: its body could be a protean landscape, that morphs in physical space to portray different information to the user. A shape-shifting landscape-robot could be well-suited for elementary education, could interact effectively with visually impaired human users, or could provide adversarial training for autonomous vehicles. The example of *The Adventures of a Hackney Coach* has already been trialled by experimental writer Ross Goodwin. His novel *1 the Road* was written by a neural network image-captioning system hooked up to a webcam that he bolted to the top of a car (Goodwin, 2018). His neural network did not meet the same embodiment and creativity criteria as Kilner’s Hackney Coach (Tables 3, 4), but it did make use of a vehicle’s embodiment as part of the creative system.

This view of creative agency has a third challenging implication. By de-emphasizing the role of general intellect and other humanlike forms of agency, these texts foreground the creative contribution of the environment or situation. The Hackney Coach composes a highly original novel by simply recording the chance encounters thrown at it by the busy metropolis of London. Coleridge and Mörike’s harps produce powerful music in collaboration with the wind. The bowling green literally *is* the environment. With their broad understanding of agency, authors such as Coleridge or Goethe found it easy to explain how the environment itself could be creative. An interesting challenge, ripe for further exploration in creative robotics, is therefore to model how the physical environment has input into the creative process, as reflected on by the “Press” (environment) variable being one of the *Four Ps* of creativity (Rhodes, 1961; Jordanous, 2016). Some useful steps forwards in this area have already been taken, e.g. Saunders’ *Curious Whispers* embodied interactive creative agents (Saunders, 2012), inspired by Csikszentmihalyi’s systems approach to creativity (Csikszentmihalyi, 1988); and Jon McCormack’s Eden project (McCormack, 2001), an artificial ecosystem where inhabitants have a creative interactive co-evolutionary relationship with their environment. We do also however acknowledge the alternative perspective of behaviour-based robotics and related Artificial Life research, that advocates a largely representation-free interaction with the external physical environment (Brooks, 1991; Jordanous, 2020).

### 4.2 An Exploration of Ethics

So far we have considered these fictional examples as possible designs which scientists could evaluate or emulate. But design fictions can also serve another purpose: to help “us to explore ethical and social issues within the context of everyday life” (Dunne and Raby, 2013, p. 51). Our fictional examples reveal some of the risks posed by creative AIs, and may help scientists anticipate unintended consequences of their research. Kilner’s Hackney Coach overhears the private conversations of its passengers without their knowing. Goethe’s Homunculus is able to read human thoughts. On a slightly different tack, Hoffmann’s Talking Turk is built to conceal its operations, and to appeal to racist stereotypes about Oriental mysticism; in these ways the Turk’s creator uses it to prey upon paying customers, allowing its creator to control and profit from the public (see Falk (2021)). In each of these cases, the embodiment of the artificial agent affects how humans interact with it, with potentially disastrous results. Some of the examples are chillingly relevant today. Cars, phones and home assistants all have the capacity to record their users, and often do: What is to stop them spilling the beans, as the Hackney Coach does, particularly as text generation improves? The Talking Turk, meanwhile, offers a critical perspective on the design of chatbots and other question-answering systems. How might artificial voices or faces be designed, and how can consumers and the public be protected from subconscious manipulation?

Homunculus presents a special case. His peculiar embodiment gives him peculiar capabilities. Like the superintelligent AIs of contemporary cyberpunk novels, he seems to inhabit a world of pure information. Though he is able to hear and speak, he also emits light that grants him direct access to other characters’ minds. Shortly after coming into existence, he levitates over the sleeping character Faust, and observes his dreams:

Homunculus (erstaunt)

Bedeutend! –

(*Die Phiole entschlüpft aus Wagners Händen, schwebt über Faust und beleuchtet ihn.*)

Schön umgeben!—Klar gewässer

Im dichten haine, Frau’n die sich entkleiden […] (Goethe, 1832, p. 107, p. 107)

[Homunculus (astounded)

Remarkable! –

(*The phial slips out of Wagner’s hands, floats over Faust and illuminates him.*)

Such beautiful scenery!—Clear water

In the shady grove, women undressing themselves […]]

Through this direct mind-machine interface, Homunculus is able to peer into Faust’s erotic dreams, which the sleeping professor would surely have preferred to keep private. The character Mephistopheles links Homunculus’s mind-reading capabilities to his embodiment: “So klein du bist, so groß bist du Phantast” [So small you are, yet such a great fantasist.] (Goethe, 1832, p. 108). The word “Phantast” is crucially ambiguous, meaning something between “dreamer” and “novelist”. There seems to be a relationship between Homunculus’s tiny presence and fragility in the physical world, and his intimidating presence and creativity in the psychic world. In the end, Homunculus forsakes both his physical presence and self-identity altogether, when he fuses with Proteus in a flash of light (Goethe, 1832, p. 178). His choice of Proteus to fuse with is highly significant: Proteus is a shapeshifting Greek deity of the sea, whose body never remains in the same form for more than an instant. In some ways, Homunculus seems to foreshadow cyberpunk fantasies about uploading the mind into the cloud and living a virtual, disembodied life.

At first glance, Homunculus may seem barely credible as an AI design, but in fact he exhibits crucial properties of actually existing systems. Many contemporary AI systems exist primarily as software, and lack embodiment in much the same way as Homunculus. Likewise, Mephistopheles is quite right to suggest that Homunculus’s diminutive embodiment is linked to his mind-reading abilities. Like Homunculus, devices such smartphones, home assistants, cochlear implants or Elon Musk’s neuralink chip all need to be small in order to penetrate human lives or human brains. Siri and Alexa may actually reside in giant data centres, but in users’ day-to-day lives they take the form of small, limbless, glowing bodies, and persistently monitor users’ behaviour in order to read (or rather, model) their minds. Both Homunculus and the Hackney Coach take advantage of their embodiment to slip into human lives. In both *Adventures of a Hackney Coach* and *Faust*, the results are creative and positive: the Coach produces a brilliant satirical novel based on its secret observations, while Homunculus develops a higher form of consciousness and merges with Proteus. But the darker ethical implications are there to see, and may provoke important discussions in laboratories and design studios.

### 4.3 The Method of Historical Design Fiction

We conclude this discussion by reflecting on the multi-pronged approach to analysis adopted in this research. The Design Fictions approach enabled us to treat historical fiction as sources of inspiration for future creative robotics research. Our decision to use componential characterisations of creativity and embodiment has given us a multi-dimensional model by which to examine the historical texts. By considering so many different aspects of creativity and embodiment in a systematic manner, we could highlight many new and useful details to bring forward from the 18th Century to present-day attention, and we could consider examples that may not have fulfilled the requirements of the more rigid ‘bifold’ definition of creativity.

## 5 Conclusion

In our work we have examined several literary depictions from the Long 18th Century (c. 1650–1850) describing different kinds of embodied systems or autonomous entities capable of contributing to human creativity. Initially, the idea of examining past fictitious examples may seem curious; however the design fictions approach reveals that these examples vividly capture some of the ideals concerning creativity support and co-creativity during the time. In particular, we see an emphasis on active involvement, directed perception, experimentation and the ability to capitalize on spontaneity, with embodiment and interaction crucial to generating valuable results.

Based on our discussion of these examples, we offer four guidelines to researchers in creative AI and robotics. These are guidelines for *discovery*. They aim to widen the search space of design and research possibilities, and give researchers a finer sense of the contours of the search surface. What kinds of artificial creative agents are possible? Which are preferable?

1. **Explore unlikely embodiments:** Eighteenth-century examples invite us to widen the space of possible mechanistic co-creative partners. In *Tristram Shandy*, one creative system has a flat body of soft soil. In Coleridge and Mörike’s eolian harp poems, the systems have resonant bodies of wood and gut. What new kinds of creative action might be enabled by other strange materials, body shapes and dimensions?

2. **Think of situations, not systems:** Our examples emphasize the way that systems draw on the environment to create new situations. The Hackney Coach creates a new series of connections between people and places in London, because of its imperceptibility and position in a network of human interactions. Inspired by such fictions, researchers can look beyond individual systems, and consider what new *situations* they would like to bring into being. What new environments or connections, what new patterns of interaction or behaviour might different embodiments bring about?

3. **Be aware of the disjunction between action and appearance:** How an agent appears can often conceal what it does. In *Faust*, Homunculous’s tiny, fragile body conceals vast capacities—indeed, Mephistopheles suggests that his tininess actually *enables* his vast capacities. Likewise the small physical footprint of a modern agent like Alexa can conceal the large distributed system it embodies, and enable that system to penetrate people’s lives. Ethical designers should consider what to conceal and what to reveal in their artificial agent’s embodiment.

4. **Consider the system as a situated moral agent:** We have seen how in many of these fictions, apparently unconscious or static agents act in self-consistent and unpredictable ways, much like conscious human agents. Uncle Toby’s bowling-green brings about Tristram’s circumcision. Hoffmann’s musical automata challenge Ferdinand and Ludwig to reconsider who is the composer of a novel tune. While it can be tempting to deny agency to mechanistic creative systems, a mechanistic system’s potential consequences come more sharply into view if we consider the system itself as the one who acts.

Overall, like the example of the musical dice, the historical ideas and inspirations we highlight above can capture the imagination of modern roboticians and co-creativity scholars, and can inspire their efforts, unhindered by any potential current technical blinders. Thus, understanding how long-18th-century authors viewed mechanical/robotic creativity offers a firm foundation for building models for modern co-creative robotics. As famously observed by Sagan, “you have to know the past to understand the present.”

## Data Availability

The original contributions presented in the study are included in the article/supplementary material, further inquiries can be directed to the corresponding author.

## Footnotes

1 The exception was *Faust*, which was read by the literary expert alone, and then discussed at length with the team.

2 This aligns with modern-day interpretations of what it means to be creative when generating music (Jordanous and Keller, 2012): social communication and interaction, domain competence and intention/emotional involvement were found to be crucial factors, and Ludwig is questioning the ability of the automatons to engage with their music intentionally and with emotion.

3 “Piss out of the window”
